# Intravenous Calcium Alginate Microspheres as Drug Delivery Vehicles in Acute Kidney Injury Treatment

**DOI:** 10.3390/mi13040538

**Published:** 2022-03-29

**Authors:** Jia Man, Xiaojie Wang, Jianyong Li, Xiaoyang Cui, Zesheng Hua, Jianfeng Li, Zebing Mao, Shanguo Zhang

**Affiliations:** 1 Key Laboratory of High Efficiency and Clean Mechanical Manufacture of MOE, School of Mechanical Engineering, Shandong University, Jinan 250061, China; mj@sdu.edu.cn (J.M.); 202014268@mail.sdu.edu.cn (Z.H.); ljf@sdu.edu.cn (J.L.); 2 Key National Demonstration Center for Experimental Mechanical Engineering Education, Shandong University, Jinan 250061, China; 3 Department of Pharmacology, School of Basic Medical Sciences, Shandong University, Jinan 250012, China; wangxiaojie@sdu.edu.cn (X.W.); 201613798@mail.sdu.edu.cn (X.C.); 4 Smart Materials Lab, Department of Engineering Science and Mechanics, Shibaura Institute of Technology, 3-7-5 Toyosu, Koto-ku, Tokyo 135-8548, Japan

**Keywords:** drug delivery, alginate microspheres, microfluidic, acute kidney injury treatment, intravenous injection therapy

## Abstract

Acute kidney injury (AKI) is a common and severe problem associated with high morbidity, mortality, and healthcare costs. There are no reliable therapeutic interventions except dialysis that could improve survival, limit injury, or speed up recovery. Thus, it is essential to develop new therapies to treat AKI. Previous studies revealed that histone deacetylase inhibitor (HDACi) could attenuate renal injury and enhance kidney recovery in AKI. However, the hydrophobic nature of HDACi, such as vorinostat (SAHA), requires organic solvents to promote its dissolution, leading to inevitable detrimental effects. Herein, calcium alginate microspheres (CAM) were prepared by the microfluidic method as HDACi carriers to treat AKI by intravenous injection. First, we designed the structure of the microfluidic channel for the fabrication of the PDMS microfluidic chip in which the emulsion state of droplets was analyzed. As the flow rate increases, the continuous phase changed from laminar flow to the dripping pattern in the microfluidic device. Then, the CAM was fabricated by a W/O microfluidic emulsion template and the size of the microspheres was adjusted from 3 to 7 μm by the concentration of alginate and the flow rate of the continuous phase and dispersal phase. The higher degree of cross-linking of sodium alginate with calcium ions would lead to longer drug release time but lower swelling rates. Furthermore, we selected CAM with suitable sizes as the HDACi carrier and delivered the HDACi-loaded CAM to the AKI mice by intravenous tail injection. The in vivo results showed that the HDACi-loaded CAM could effectively reduce the renal regional inflammatory response and attenuate renal injury.

## 1. Introduction 

Acute kidney injury (AKI) is a clinical syndrome with multiple aggressive factors, such as severe infection, sepsis, and ischemia-reperfusion. It causes a critical decline in renal function over a short period, usually characterized by decreased urine output, azotemia, disturbed fluid balance, electrolyte disturbances, metabolic acidosis, neurological, and hematologic disturbances [1]. AKI can occur in multiple clinical disciplines, and approximately 30 to 70% of surviving AKI cases could progress to chronic kidney disease, with approximately 17% of AKI patients progressing to end-stage renal disease within one year [2]. Currently, there are no pharmacotherapies approved for AKI treatment. Thus, it is essential to find effective therapies and improve the prognosis of AKI renal function. 

Histone deacetylase inhibitor (HDACi) is a kind of compound that can interfere with the function of histone deacetylase. Recent studies have shown that HDACi vorinostat (SAHA) can protect the kidney and are highly effective in the treatment of acute kidney injury [3]. However, SAHA is a kind of hydrophobic drug and cannot be dissolved in an aqueous solution. Therefore, most organic solvents, such as dimethyl sulfoxide (DMSO), are used to facilitate the dissolution of HDACi, which may cause symptoms such as pruritus, intravascular hemolysis, and gastrointestinal discomfort [4]. Unlike the direct intravenous injection of drugs dissolved in organic solvents, the encapsulation of the drug in biocompatible microspheres allows for the release of the drug, avoiding the pain caused to the patient by frequent administration and reducing the side effects caused by organic solvents. Sriram et al. demonstrated that hydrophobic drugs could be encapsulated in microspheres and administered intravenously to treat disease [5]. Sibghatullah et al. used the method of spray drying to prepare gelatin microspheres encapsulating 5-fluorouracil with the particles size range of 5–15 μm for the treatment of lung cancer by intravenous injection, and the results showed that the drug-loaded microspheres improved the release time of the drug [6]. Thus, AKI therapy by intravenous injection of SAHA-loaded calcium alginate microspheres is a promising method. However, size has a marked effect on the properties of microspheres injected into the vasculature. For intravenous use, large particles may lodge in the pulmonary vasculature, which may be a clinical problem. Researchers have found that large quantities of less than 5 μm particles could be injected directly into the blood vessel of mice without causing detectable problems [7]. Besides, microspheres with low sphericity and wide size distribution cannot achieve consistent and stable drug release profiles.

Alginate is a biocompatible material that has been extensively used for drug delivery [8,9]. The morphology of drug-loaded microspheres has an important impact on the drug release property. Compared with irregular microspheres, particles with high sphericity could have a continuous and stable drug release property [10,11]. Currently, drug-loaded particles can be prepared by different methods, such as spray drying and solvent evaporation, but these methods have the disadvantage that the morphology of microspheres cannot be precisely controlled [12]. Thus, it is necessary to prepare drug-loaded microspheres with properties of high sphericity, suitable and uniform size for the intravenous treatment of AKI. The microfluidic method can manipulate the size of droplets at the microscale and regulate the composition and morphology of microspheres precisely [13]. Based on these advantages, the microspheres prepared by microfluidics have properties of controllable size, good monodispersity, and diverse morphology [14]. In this study, we first designed a T-junction PDMS microfluidic chip. Then we investigated the emulsion pattern of the droplets inside the microfluidic chip after using 1-undecanol containing CaI_2_ as the continuous phase and the aqueous solution of alginate as the disperse phase. We studied the effect of the flow rate ratio and the concentration of alginate on the size of calcium alginate microspheres (CAM). In addition, we examined the release property of vorinostat (SAHA) from calcium alginate microspheres with different degrees of crosslinking. Intravenous microspheres should remain stable in size after injecting into the blood, so we explored the swelling property of highly crosslinked CAM. Finally, SAHA-loaded CAM was delivered to mice of AKI by tail intravenous injection to identify the therapeutic effect.

## 2. Materials and Methods

### 2.1. Materials

1-Undecanol, sodium alginate (vis 200 ± 20 mpa·s), vorinostat (SAHA), tween-80, and dimethyl sulfoxide (DMSO) were purchased from Aladdin Bio-Chem Technology Co., Ltd. (Shanghai, China). Ethyl ethanol was purchased from Sinopharm Chemical Reagent Co., Ltd. (Beijing, China). The other chemicals or reagents, unless mentioned elsewhere, were purchased from Aladdin.

All reagents were of analytical grades and used as received.

### 2.2. Size Control of CAM

The PDMS microfluidic chip was fabricated by the process of photolithography (Figure S1). Then, we established the online observation microfluidic platform for CAM preparation, as shown in Figure S2. In the microfluidic platform, the alginate solution was used as the dispersed phase and the 1-undecanol containing calcium iodide (CaI_2_) was used as the continuous phase. Based on our previous studies, CAM with high sphericity can be prepared by selecting a suitable concentration of calcium ions. The size of the CAM could be regulated by the flow rate ratio of the continuous phase to the dispersed phase. We set the concentration of CaI_2_ and sodium alginate at 0.1 wt% and 1 wt%, respectively. The flow rate of the disperse phase was fixed at 5 μL/h and the flow rates of the continuous phase were adjusted to 5 μL/h, 15 μL/h, 36 μL/h, and 90 μL/h. Using the concentration of sodium alginate could also control the size of CAM. The concentration of alginate was adjusted to 1 wt%, 0.1 wt%, 0.04 wt%, and 0.02 wt% and the flow rate of 5 μL/h in the dispersed phase, and 15 μL/h in the continuous phase. The CAM was observed by scanning electron microscopy (SEM, JSM-6610LV, Nippon Electronics Co., Tokyo, Japan). Image J software was used to calculate the size distribution of CAM.

### 2.3. Drug Loading and Release 

The CAM were prepared by the microfluidic chip in the 1-undecanol solution containing 0.1 wt% CaI_2_. We prepared three groups of CAM of high sphericity with average diameters of about 3 μm. Afterward, the three groups of CAM were immersed in ethanol solutions containing 2 wt%, 5 wt%, and 10 wt% of CaCl_2_ for 24 h to finish the final cross-linking. Vorinostat (SAHA) is a kind of hydrophobic drug, which can be dissolved by dimethyl sulfoxide (DMSO). To reduce the use of the cytotoxic organic solvent DMSO, we used a compound solvent to dissolve vorinostat (SAHA). The composition was PEG300:water:Tween 80:DMSO = 30:61:5:4. We immersed 600 μg CAM in 0.5 mg/mL SAHA solution for 24 h to obtain SAHA-loaded CAM. The absorbance of the SAHA solution with different concentration was measured using a UV spectrophotometer (Agilent, Cary 100, Santa Clara, CA, USA) and the standard curve was listed as follow:

$$Y=4.6663\;X+0{.875\;R}^{2}=0.9964$$

The SAHA-loaded CAM was placed in a cuvette with 4 mL of phosphate buffer and the absorbance values of SAHA were measured at special times until the absorbance did not change. Each group of the experiment was repeated three times.

### 2.4. Swelling Property

The CAM was crosslinked in ethyl alcohol containing 10 wt% CaCl_2_ for 24 h. They were then washed three times with pure ethyl alcohol and immersed in phosphate buffer (pH = 7.4) for 2 h. After quick freezing in liquid nitrogen and freeze-drying in a lyophilizer, the CAM could be observed by SEM for their size and morphology after swelling.

### 2.5. Mice Surgical AKI Model

The animal experiment was approved by the Animal Protection Committee of Shandong University, and all the operating procedures were conducted according to the National Institutes of Health Guide. The AKI model was established via ischemia-reperfusion surgery in mice. C57/B6 mice (6–8 weeks) were randomly divided into 4 groups: healthy mice (control), AKI model (IRI), AKI model treated with SAHA by tail vein injection (IRI/SAHA), and AKI model treated with SAHA-loaded CAM by tail vein injection (IRI/SAHA/microspheres). 

SAHA was dissolved in the solvent of 4% DMSO, 30% PEG300, 5% Tween80, 61% H_2_O, and the final concentration of SAHA was 2.5 mg/mL. The final dose of SAHA injected into mice was 20 mg/kg through the tail vein; CAM with an average diameter of about 3 μm was immersed in SAHA solution for 24 h to obtain the same dose of SAHA as described above. The SAHA-loaded CAM was also delivered into mice by tail vein injection.

## 3. Results and Discussions

### 3.1. Emulsion Patterns in Different Flow Rate

**Figure 1:**
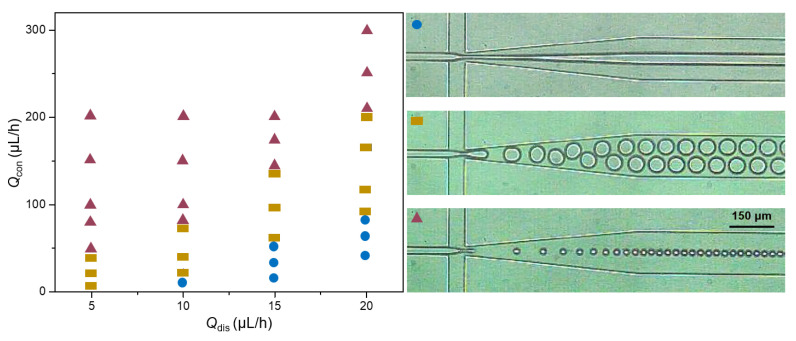
Emulsion patterns of dispersed phases at different flow rates.

The emulsion state of alginate in microfluidic chips is highly dependent on the flow rates of the continuous phase (*Q*_con_) and the dispersed phase (*Q*_dis_*)*. The dispersed phase could be sheared into droplets due to the combined effect of viscous forces and interfacial tension from the continuous phase [15]. However, when the flow rate of the continuous phase decreased, the surface tension between the two phases was less than the inertial force of the dispersed phase, and the dispersed phase could not be sheared into droplets, resulting in laminar flow in the microfluidic channel (Figure 1, circles) [16]. When the flow rate of the two phases was suitable for the dripping pattern, we could obtain uniform droplets (Figure 1, rectangles). When we increased the flow rates of the continuous phase, the disperse phase could still be sheared into droplets with smaller sizes (Figure 1, triangles) [17]. 

### 3.2. Control of the Size of Microspheres

**Figure 2:**
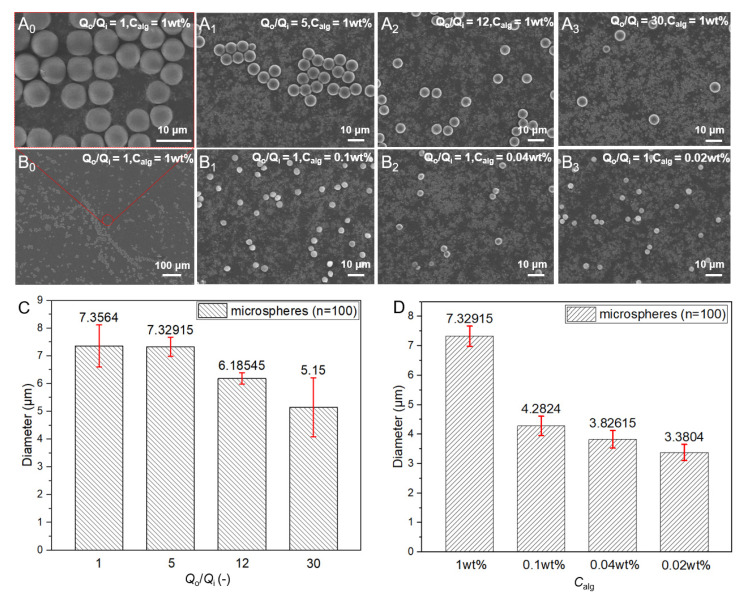
Size control of CAM by adjusting the flow rate ratio between continuous and dispersed phases (**A_0–3_**) and the concentration of alginate (**B_0–3_**) with corresponding size calculation (**C**,**D**), respectively.

The content of alginate in the droplets could determine the size of the microspheres as the water in the droplet would escape in the gelation process until the final solidification. Thus, the size of the CAM could be adjusted by the concentration of alginate and the flow rate ratio of the continuous phase to the dispersed phase. As shown in Figure 2A_0_ and B_0_, when the concentration of alginate was 1 wt% and the flow rates of the dispersed and continuous phases both were 5 μL/h, we could get monodisperse CAM microspheres with an average diameter of approximately 7.3 μm (Figure 2C). As shown in Figure 2A_0–3_,C, when the concentration of alginate remained constant and the flow rate ratio between continuous and dispersed phases was gradually increased, it was found that the size of CAM gradually decreased. Besides, the flow rate ratio increased by 30 times while the average size only decreased by about 2 μm. When the flow rate ratio was fixed, the size of the CAM decreased as the concentration of the alginate decreased, as shown in Figure 2B_0–3_,D. The results showed that the size of the CAM was rarely affected when the concentration of alginate was too low, from 0.1 wt%, 0.04 wt% to 0.02 wt%. Thus, we could control the size of the CAM range from about 3 to 7 μm. 

### 3.3. Drug Release Properties of CAM

**Figure 3:**
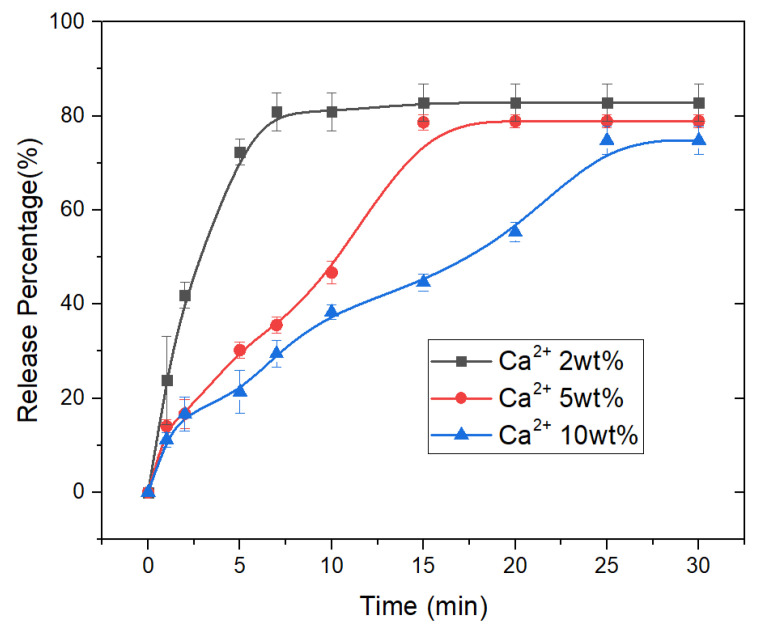
Drug release profiles of CAM after crosslinking with different concentrations of calcium ions.

To decrease the size of the microspheres, the concentrations of alginate and Ca^2+^ used were both low, resulting in incomplete crosslinking of alginate in the microspheres. Thus, these microspheres could not be used directly as drug carriers. Considering the requirement of intravenous injection for drug-loaded microspheres, we investigated the drug release property of CAM with average diameters of about 3 μm after being crosslinked with different concentrations of calcium ions. As shown in Figure 3, the drug release time gradually increased as the concentration of Ca^2+^ increased. When the crosslinked calcium ion concentration was 2 wt%, the drug release time was only about 7 min. However, when the calcium ion concentration increased to 10 wt%, the drug release time was extended to about 25 min. The CAM crosslinked with low concentrations of calcium ions had a sudden drug release property in the initial phase, and the drug release profiles gradually stabilized as the crosslinking strength increased.

### 3.4. Swelling Properties of CAM

**Figure 4:**
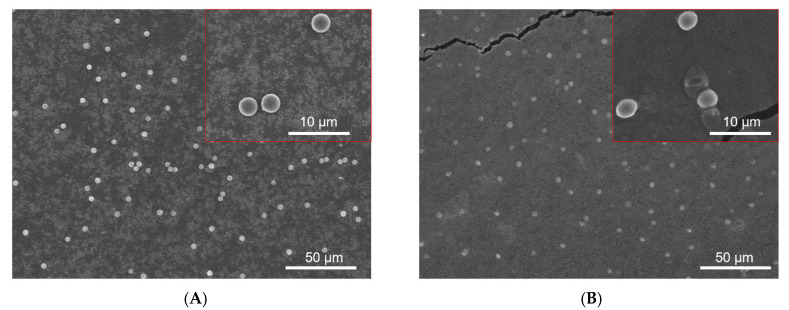
Swelling properties of Ca-ALG microspheres. The SEM images of Ca-ALG microspheres crosslinked with Ca^2+^ (10 wt%) before (**A**) and after (**B**) swelling.

The CAM has the property of swelling in aqueous solutions, resulting in size enlargement. For intravenous administration, microparticles of large size would be harmful to the human body. Thus, ideal drug-loaded microspheres for intravenous administration should have suitable sizes and swelling rates after swelling. Besides, the drug release profiles above showed that CAM after being crosslinked by calcium ion concentration of 10 wt% had the longest release time. Therefore, we studied the swelling property of that CAM. As shown in Figure 4, the size change of the CAM was very little after swelling, indicating that the microspheres could maintain stable morphology in the blood.

### 3.5. Drug Loaded CAM for AKI by Intravenous Injection

**Figure 5:**
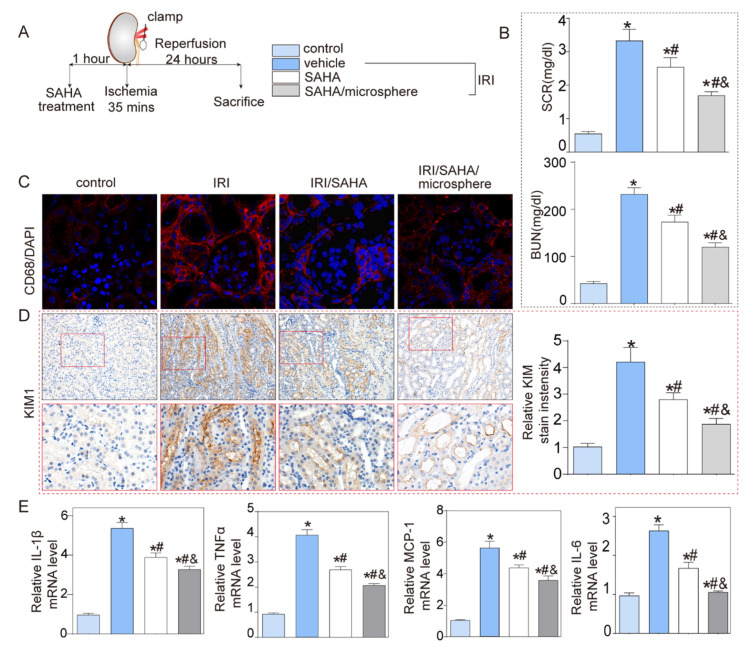
SAHA-loaded CAM ameliorated IRI injury. (**A**) Methods for AKI model and microspheres delivery. (**B**) SAHA and SAHA-loaded CAM increased renal function by decreasing BUN and SCR levels, respectively. (**C**) The fluorescent staining of CD 68 proteins. (**D**) Immunohistochemistry staining (IHC) staining of CD68 and relative CD68 positive cell/field. (**E**) The mRNA levels of IL-1β mRNA, TNF-α mRNA, MCP-1 mRNA, and IL-6 mRNA in 4 groups, respectively. Data presented as mean ± SEM and analyzed by one-way ANOVA. * *p* < 0.05 versus vehicle, # *p* < 0.05 versus IRI mice and & *p* < 0.05 versus SAHA treatment mice. n = 6.

In the present study, we used a microfluidic method to generate CAM with the size range from about 3 to 7 μm, and it was demonstrated that the microspheres were suitable for intravenous injection. To evaluate the in vivo curative effect of HDACi-loaded microspheres, we established animal models of AKI in mice for three groups by ischemia-reperfusion injury as shown in Figure 5A, and one healthy group of mice were used as a control group. The mice received treatment with either SAHA or SAHA-loaded microspheres. Serum blood urea nitrogen (BUN) and serum creatinine (SCR) are two important indicators of kidney function. In general, the degree of renal injury increased with the increase in BUN and SCR values. As shown in Figure 5B, BUN and SCR levels significantly increased in the IRI group comparing with the healthy groups, but the values always were the lowest in the SAHA-loaded microspheres-treated groups. Thus, microspheres could improve the therapeutic effect of HDACi. CD68 is a protein highly expressed by the macrophage. As shown in Figure 5C, we used DAPI-labeled CD68 to identify macrophages in tissue sections. To figure out the changing of cytokines which can reflect the protective capability of microspheres according to suppression of inflammation, we designed the immunohistochemistry staining experiment. As shown in Figure 5D, compared with the SAHA group, the SAHA-loaded microspheres group significantly reduced macrophage infiltration, which could account for the amelioration of inflammatory response. The higher relative levels of the four inflammatory cytokines—IL-6 mRNA, IL-1β mRNA, TNF-α mRNA, and MCP-1 mRNA—represented a more intense inflammatory response. As shown in Figure 5E, the relative levels of inflammatory cytokines were all lowest in mice groups treated by injection of SAHA-loaded CAM compared to the SAHA group, indicating that the microspheres could effectively inhibit the inflammatory response and reduce the side effects of the drug.

## 4. Conclusions

In conclusion, we fabricated CAM with the size of about 3 μm using a W/O microfluidic emulsion template method to deliver HDACi for AKI treatment. By adjusting the concentration of alginate and the flow rate of the continuous phase and dispersal phase, we obtained the monodisperse microspheres with size ranging from about 3 to 7 μm. After being crosslinked with different concentrations of Ca^2+^, the CAM has the property of controllable drug release and suitable swelling. Then, the SAHA-loaded CAM was applied in vivo AKI treatment by intravenous injection. It was demonstrated that the CAM was effective in AKI treatment, avoiding the toxic and side effects caused by the organic solvent. Thus, the HDACi-loaded CAM combined with intravenous injection therapy might be a promising method for AKI treatment.

## Data Availability

The data presented in this study are available on request from thecorresponding author. All of the raw data are not presented due to their type and large number.

## Supplementary Materials

The following are available online at https://www.mdpi.com/article/10.3390/mi13040538/s1, Figure S1: Structure of the microfluidic channel., Figure S2: (A) Online observation microfluidic platform for CAM preparation. (B) T-shaped PDMS microfluidic chip. (C) Optical images of sodium alginate droplets.
