# Supplementary material for: Intravenous Calcium Alginate Microspheres as Drug Delivery Vehicles in Acute Kidney Injury Treatment
Source: Micromachines (Basel). 2022 Mar 29;13(4):538. doi: 10.3390/mi13040538 (PMC9026119; doi:10.3390/mi13040538)
Supplement: Supplementary file 1 [file micromachines-13-00538-s001.zip › micromachines-1655909-supplementary.pdf]

micromachines

MDPI

Article

Intravenous Calcium Alginate Microspheres as Drug Delivery Vehicles in Acute Kidney Injury Treatment

Jia Man [1,2,†], Xiaojie Wang [3,†], Jianyong Li [1,2,*], Xiaoyang Cui [3], Zesheng Hua [1,2], Jianfeng Li [1,2], Zebing Mao [4,*] and Shanguo Zhang [1,2,*]

1 Key Laboratory of High Efficiency and Clean Mechanical Manufacture of MOE, School of Mechanical Engineering, Shandong University, Jinan 250061, China; mj@sdu.edu.cn (J.M.); 202014268@mail.sdu.edu.cn (Z.H.); ljf@sdu.edu.cn (J.L.)
2 Key National Demonstration Center for Experimental Mechanical Engineering Education, Shandong University, Jinan 250061, China
3 Department of Pharmacology, School of Basic Medical Sciences, Shandong University, Jinan 250012, China; wangxiaojie@sdu.edu.cn (X.W.); 201613798@mail.sdu.edu.cn (X.C.)
4 Smart Materials Lab, Department of Engineering Science and Mechanics, Shibaura Institute of Technology, 3-7-5 Toyosu, Koto-ku, Tokyo 135-8548, Japan
* Correspondence: ljy@sdu.edu.cn (J.L.); zebingv5@shibaura-it.ac.jp (Z.M.); zsggg@mail.sdu.edu.cn (S.Z.)

Citation: Man, J.; Wang, X.; Li, J.; Cui, X.; Hua, Z.; Li, J.; Mao, Z.; Zhang, S. Intravenous Calcium Alginate Microspheres as Drug Delivery Vehicles in Acute Kidney Injury Treatment. *Micromachines* **2022**, *13*, 538. https://doi.org/10.3390/mi13040538

Academic Editor: Anna Vikulina

Received: 12 March 2022
Accepted: 28 March 2022
Published: 29 March 2022

Publisher's Note: MDPI stays neutral with regard to jurisdictional claims in published maps and institutional affiliations.

Copyright: © 2022 by the authors. Licensee MDPI, Basel, Switzerland. This article is an open access article distributed under the terms and conditions of the Creative Commons Attribution (CC BY) license (https://creativecommons.org/licenses/by/4.0/).

Fabrication of PDMS Microfluidic Chip

The width of the microfluidic channel needs to be as close as possible to the size of the target microsphere and the resistance to the flow of the fluid in the microchannel should be minimized. Thus, a T-junction microfluidic channel was designed using AutoCAD software, as shown in **Figure S1.** A mold containing the above microfluidic channel was then fabricated using photolithography, and the height of the channel was designed to be 25 μm.

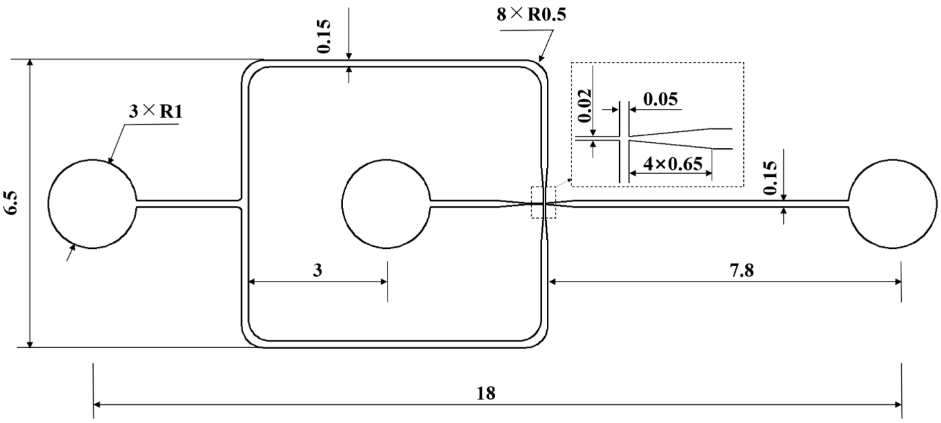

Figure S1. Structure of the microfluidic channel.

The steps for the preparation of microfluidic chips are as follows: (1) The polydimethylsiloxane (PDMS) was mixed with a curing agent in a ratio of 10:1 and then be poured evenly on the mold. (2) air bubbles in the PDMS were removed by vacuum treatment and we cured the PDMS at 75°C for 2h. (3) The PDMS layer with microfluidic channels was processing by cutting and punching. (4) A uniform layer of PDMS was applied to the slide using a glue homogenizer and cured in the oven at 120°C for 0.5h. (5) We used the ion gun to treat the side of the PDMS with the channel and the PDMS on the slide for the 30s. (6) The two sides were lightly pressed together and treated at 120°C for 2 h to obtain a bonded PDMS microfluidic chip.

The disperse and continuous phases were loaded into an independent injector driven by syringe pumps (LongerPumpLSP01-3A). The injectors were connected to the PDMS microfluidic chip prepared above via a Teflon capillary tube as shown in **Figure S2.**

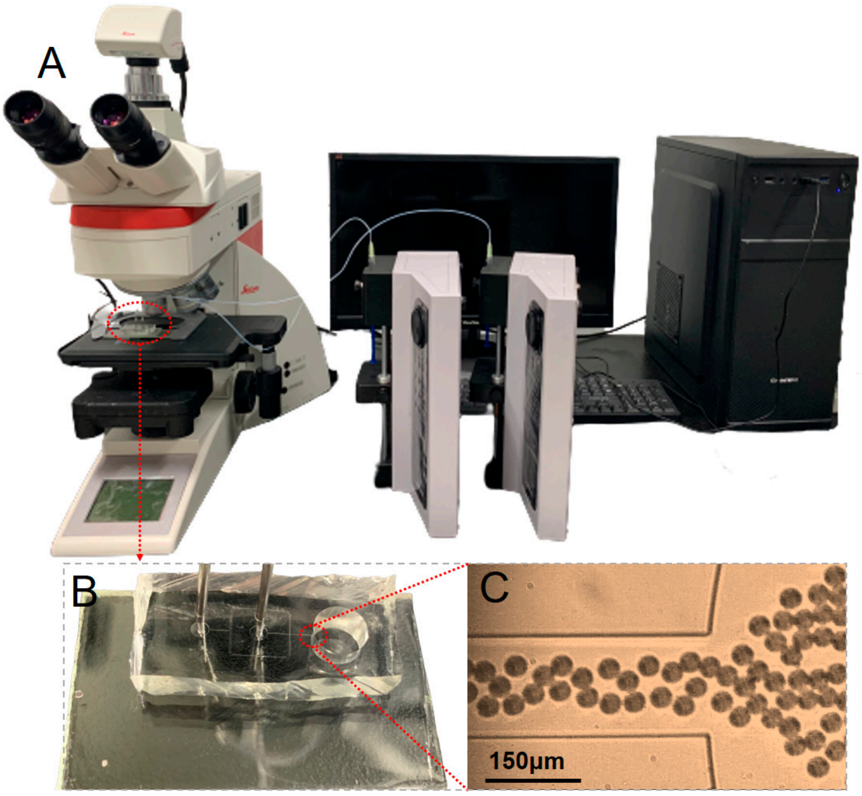

Figure S2. (A) Online observation microfluidic platform for CAM preparation. (B) T-shaped PDMS microfluidic chip. (C) Optical images of sodium alginate droplets.
